# *Smilax sinovietnamensis*, a New Quadrangular Stem Species of Smilacaceae Identified Through Integrative Taxonomy

**DOI:** 10.3390/plants15193024

**Published:** 2026-10-02

**Authors:** Jiamin Wang, Haoran Xu, Tongtong Zhang, Shenglu Zhang, Jing Wu, Yan Yu, Zhechen Qi, Chengxin Fu, Ang Liu, Qing Ma, Xinjie Jin, Pan Li

**Affiliations:** 1 College of Life and Environmental Science, Wenzhou University, Wenzhou 325035, China; 2 Zhejiang Provincial Key Laboratory of Water Ecological Environment Treatment and Resource Protection, Wenzhou University, Wenzhou 325035, China; 3 Agricultural and Rural Bureau of Lin’an District, Hangzhou 311300, China; zslwantee@126.com; 4 Laboratory of Systematic & Evolutionary Botany and Biodiversity, College of Life Sciences, Zhejiang University, Hangzhou 310058, China; 12307018@zju.edu.cn (J.W.); cxfu@zju.edu.cn (C.F.); 5 Key Laboratory of Bio-Resources and Eco-Environment of Ministry of Education, College of Life Sciences, Sichuan University, Chengdu 610065, China; yyu@scu.edu.cn; 6 Zhejiang Province Key Laboratory of Plant Secondary Metabolism and Regulation, College of Life Sciences and Medicine, Zhejiang Sci-Tech University, Hangzhou 310018, China; zqi@zstu.edu.cn; 7 Hunan Botanical Garden, Changsha 410116, China; plantorliuang@163.com; 8 College of Biology and Environmental Engineering, Zhejiang Shuren University, Hangzhou 310015, China; maqing90@live.cn

**Keywords:** morphological trait, Old World, phylogeny, Sino-Vietnamese, taxonomy, Smilacaceae, plastome

## Abstract

A new species, *Smilax sinovietnamensis* P. Li & X.J. Jin, sp. nov. (Smilacaceae), from the Sino-Vietnamese Border, is described and illustrated. Using nuclear ribosomal DNA (nrDNA), chloroplast DNA (cpDNA), and orthologous nuclear genes (ONGs), we examined the phylogenetic position of this species within the family Smilacaceae. Furthermore, phylogenetic analyses of concatenated sequences of nrITS, five cpDNA (*ndhA*, *ndhF*, *rpl16*, *matK*, and *rbcL*), and two ONGs (*At103* and *Agt1*) from 37 Smilacaceae species all strongly supported that *S. sinovietnamensis* is sister to *S. cocculoides*. Further comparative morphological analysis showed that *S. sinovietnamensis* can be clearly distinguished morphologically from its closely related species *S. cocculoides*. It has quadrangular stems and an abscission zone at one-third of the petioles’ length. Combining detailed morphological comparisons and molecular evidence, we confirm the novelty of *S. sinovietnamensis*. Additionally, we report the plastome characteristics of *S. sinovietnamensis*, enriching our understanding of the evolutionary relationships within Smilacaceae. Collectively, integrating diagnostic morphological traits with nrITS, cpDNA, ONGs, and the newly assembled plastome not only confirms *S. sinovietnamensis* as a new species but also offers practical tools for resolving taxonomic challenges in Smilacaceae. Additionally, the species’ unique quadrangular stem—an evolutionary morphology—provides significant reference value for understanding angiosperm evolution.

## 1. Introduction

Plants of the family Smilacaceae possess significant ecological, economic, and landscape values. *Smilax* L., presently recognized as the only genus within the Smilacaceae [1], stands out as one of the most abundant and distinctively adaptable climbers within the forest ecosystems across both the Old and the New World [2,3]. Commonly, they exhibit a range of habitats including woody or herbaceous vines that climb, as well as erect shrubs [4]. They possess tuberous rhizomes, with stems and branches that are often armed with prickles. Smilacaceae are notable for their uncommon dioecious nature among angiosperms, featuring unisexual flowers with six tepals and six fertile stamens or staminodes in females [5]. Qi et al. classified Smilacaceae into four major clades [2,3]: Clade A contains *S. aspera* L.; Clade B consists of mostly American species; Clade C is made up of prickless herbs, non-climbing species, and *Heterosmilax*, predominantly Asian; and Clade D includes Asian–Australian–African woody species, with Clades C and D being primarily Old World species. Following Qi et al.’s framework, in 2012 Li further detailed the Asian Smilacaceae into four subgenera and 20 sections, a classification upheld by Jin in 2017 revision [6].

Species delimitation within *Smilax* remains notoriously difficult. Substantial intraspecific morphological variation exists in many species. For example, *Smilax aspera* shows great diversity in leaf shape across its broad geographic range. Another major obstacle is that fertile materials (flowers and fruits) are hard to obtain during field surveys. Many herbarium specimens only contain vegetative parts, and some published new species were described without complete fertile materials, which increases the difficulty of accurate identification. Many closely related taxa share highly convergent vegetative traits, such as leaf shape, venation and climbing habit. Vegetative characters alone are frequently insufficient for reliable species identification. Considering these complexities, a detailed morphological revision is essential for accurate classification and identification of *Smilax* species [2,3,6,7,8,9,10,11,12,13,14,15,16,17].

Guangxi in southwestern China harbors exceptionally high plant diversity, especially within karst limestone landscapes. This region features a typical subtropical monsoon climate with abundant seasonal rainfall, high humidity and heterogeneous microhabitats formed by limestone outcrops. Karst terrains often promote population isolation and allopatric speciation, resulting in continuous discoveries of new vascular plant species [18]. Although floristic inventories have been repeatedly carried out across Guangxi, remote karst valleys remain insufficiently explored, and many cryptic or narrowly endemic plant species await formal taxonomic description [19]. Based on specimen records of this putative new species, we conducted line-transect reconnaissance surveys at these documented localities and their surrounding areas for field verification and additional sampling of *S. sinovietnamensis* (the putative new *Smilax* described below). Our field observations show that individuals of this taxon can be encountered at an approximate interval of 10 m within its suitable microhabitat, even though this species does not form dominant populations across broader areas.

During fieldwork between 2020 and 2022, we collected multiple sets of specimens of this taxon from Mianliang Village, Yongle Village and Nonghua Village of Napo County, as well as Diding Village of Nanpo Township, Jingxi City, Guangxi. Morphological comparisons revealed that this entity is most similar to *S. cocculoides* Warb., yet clearly distinguishable by its quadrangular stems and the abscission zone at one-third of the petiole length. These diagnostic morphological features separate it from all other known con-generic species. This discovery enriches the species diversity of *Smilax* and further demonstrates the remarkable biodiversity of Guangxi. Nevertheless, given the intricate taxonomic boundaries within this genus, molecular evidence is still required to firmly establish the phylogenetic placement of *S. sinovietnamensis* within *Smilax*.

Here, we describe and illustrate the new species *S. sinovietnamensis* and assemble its nuclear ribosomal DNA (nrDNA), chloroplast DNA (cpDNA) and orthologous nuclear genes (ONGs). The primary objective of this study is to formally establish this *Smilax* from the Sino-Vietnamese border region as a new taxon by integrating morphological evidence and multi-locus molecular data. To accomplish this objective, we specifically aim to (a) elucidate the morphological characteristics of the new species, *S. sinovietnamensis*; (b) explore the phylogenetic position of *S. sinovietnamensis* within the genus *Smilax*; (c) determine the size range and structure of the *S. sinovietnamensis* plastome. These results will provide valuable and basic sequence information for *Smilax* taxonomic studies and yield new insights into the identification of *Smilax* species.

## 2. Results

### 2.1. Taxonomic Treatment

**Figure 1:**
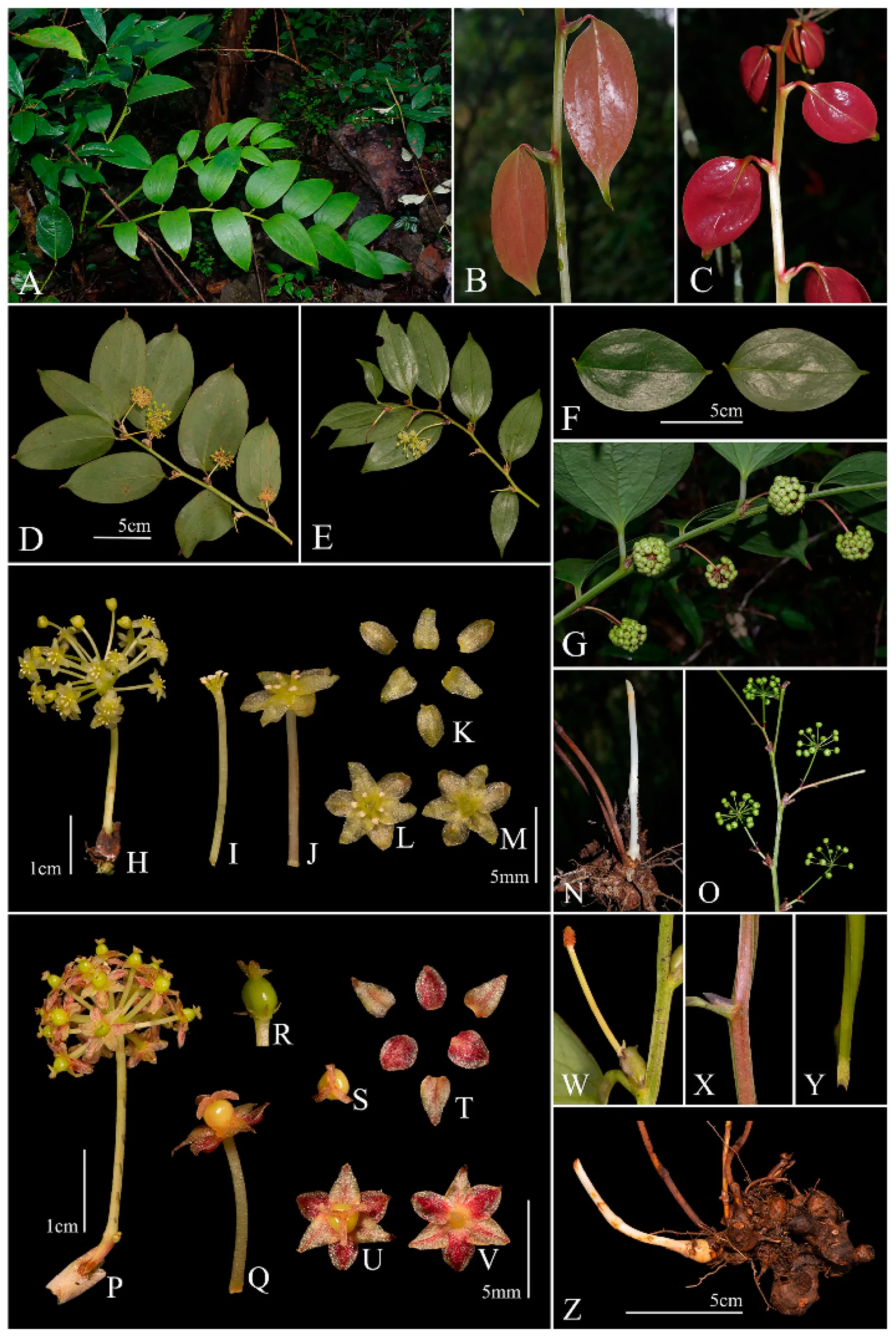
*Smilax sinovietnamensis* P. Li & X.J. Jin, sp. nov. (**A**) Plant habit; (**B**,**C**) the emerging young leaves are red, ovate to long-ovate with caudate to acuminate tips and cuneate to rounded bases; (**D**) a plant with pistillate flowers in full bloom; (**E**) plant that is blooming with a staminate flower; (**F**) adaxial and abaxial views of a plant leaf; (**G**) immature fruit; (**H**) staminate inflorescence; (**I**) staminate pedicel and stamen in side view; (**J**) staminate flower in side view; (**K**) staminate flower tepals in front view and back view; (**L**) staminate flower in front view; (**M**) staminate flower in back view; (**N**) rhizome and its sprouting new tender stem; (**O**) umbel; **(P**) pistillate inflorescence; (**Q**) pistillate flower in side view; (**R**) pistil in side view; (**S**) pistil in front view; (**T**) pistillate flower tepals in front view and back view; (**U**) pistillate flower in front view; (**V**) pistillate flower in back view; (**W**) inflorescence of 1 umbel, borne in the leaf axil, basally prophyllate; (**X**) leaf sheaths and reduced tendrils; (**Y**) quadrangular stem; (**Z**) swollen stored rhizome.

**Figure 2:**
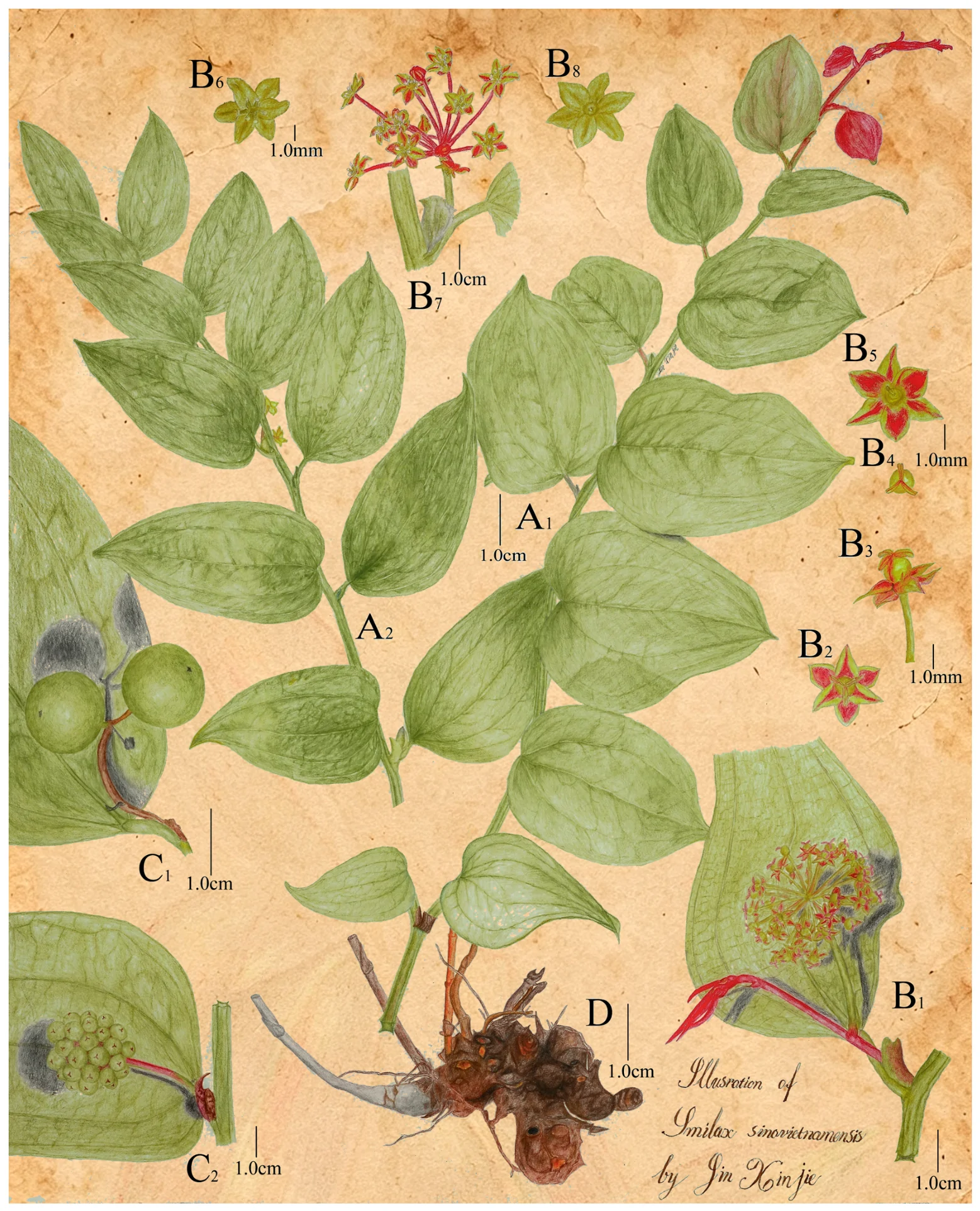
Illustration of *Smilax sinovietnamensis* P. Li & X.J. Jin, sp. nov. (**A_1_**,**A_2_**) Whole plant; (**B_1_**) pistillate flower at anthesis; (**B_2_**) adaxial view of pistillate tepals; (**B_3_**) pistillate flower in side view; (**B_4_**) pistil in front view; (**B_5_**) abaxial view of pistillate tepals; (**B_6_**) adaxial view of staminate tepals; (**B_7_**) staminate flower at anthesis; (**B_8_**) abaxial view of staminate tepals; (**C_1_**) immature fruit; (**C_2_**) umbellate infructescence; (**D**) rhizome. A miniature colored pencil drawing by Jin Xin Jie, rendered with Faber-Castell Polychromos and Tombow oil-based colored pencils.

*Smilax sinovietnamensis* P. Li & X.J. Jin, sp. nov. (中越菝葜 Zhongyue Baqia, Figure 1 and Figure 2).

Type: China, Guangxi Zhuang Autonomous Region, Baise City, Napo County, Baixing Town, Mianliang Village, Nongmiao, roadside in mountain slope forest, 23.1594007° N, 105.5750778° E, altitude ca. 1210 m, 2020 August 21, P. Li et al. WZM928. Holotype and isotypes deposited at WZUH.

Holotype: collection No.: P. Li et al. WZM928–2, barcode No.: Xin-Jie Jin00661.

Isotypes: P. Li et al. WZM928–1 (Xin-Jie Jin00675), P. Li et al. WZM928–3 (Xin-Jie Jin00662), P. Li et al. WZM928–4 (Xin-Jie Jin00660), P. Li et al. WZM928–5 (Xin-Jie Jin00663), P. Li et al. WZM928–6 (Xin-Jie Jin00664), P. Li et al. WZM928–7 (Xin-Jie Jin00665), P. Li et al. WZM928–8 (Xin-Jie Jin00666), P. Li et al. WZM928–9 (Xin-Jie Jin00667).

Paratypes: P. Li et al. PanLi009060 (Xin-Jie Jin00676), P. Li et al. PanLi009020 (Xin-Jie Jin001416), P. Li et al. PanLi010014 (Xin-Jie Jin001405), P. Li et al. PanLi010047 (Xin-Jie Jin001658), P. Li et al. PanLi009037 (Xin-Jie Jin001649) (WZUH); L. Wu 3609 (BNU 0033180) (Herbarium of College of Life Sciences, Beijing Normal University (BNU); D.X. Nong et al. 451026150526013LY (GXMG 0115765), X.Y. Huang et al. 451026131127039LY (GXMG 0115765) (Herbarium of Guangxi Botanical Garden of Medicinal Plants (GXMG); D. Fang et al. 79852 (GXMI 029956), D. Fang et al. 78458 (GXMI 029957), D. Fang et al. 79853 (GXMI 029959), Z.G. Wang 3–1700 (GXMI 029960) (Herbarium of Guangxi Institute of Chinese Medical and Medicine Sciences (GXMI); H.N. Qin 451 (1287002), H.N. Qin 509 (1291612) (Herbarium of Kunming Institute of Botany, Chinese Academy of Sciences (KUN); N.D. Hua 1040 (02017843), H.N. Qin 509 (02018132), P. J. Cribb et al. ASBK 223 (02018202) (Herbarium of Institute of Botany, Chinese Academy of Sciences (PE).

Perennial woody evergreen shrubs. Stems quadrangular, unarmed. Rhizome woody, tuberous-swollen, irregularly nodular, brown and rough on surface; nodes conspicuous, bearing numerous slender fibrous adventitious roots. New buds sprout from rhizome nodes, developing into milky-white to pale yellowish-white young creeping segments, which further differentiate into aerial climbing stems; remnants of old stems persist on rhizome nodes. Petioles 0.64–2.82 cm long, narrowly winged for about 1/5–1/2 of their length, verruculose, abscission zone at 1/3. Leaf blade coriaceous, leaf base basifixed, ovate, 6.8–19 cm long, 2.7–10.6 cm wide, with acuminate apex, base cuneate or truncate, three primary and two marginal veins. The leaf is green above and glossy beneath, pale greenish to dark cadmium orange at youth. Inflorescences of 1–2 umbels, borne in leaf axils, with prophylls at the base, hidden on the abaxial side of leaves at maturity. Peduncles 1.1–5.5 cm long, erect, slightly compressed; each umbel 10–31-flowered, hemispherical or spherical, base slightly thickened, subglobose; floret pedicels 2.0–16.1 mm long. Pistillate flowers red-yellowish; tepals six, in two whorls, deltoid to ovate, 1.4–2.4 mm long, 0.5–1.9 mm wide, glabrous on both surfaces; staminodes six; ovary superior. Staminate flowers yellow-greenish or red-yellowish; tepals six, in two whorls, 0.8–4.9 mm long, 0.3–2.9 mm wide, deltoid to ovate, inner ones slightly narrower, outer and inner ones connate for ca. 1/5; stamens six, with yellowish anthers on short filaments, two anther sacs obvious. Berries 2.41–5.92 mm in diameter, presumably dark indigo at maturity (based on GXMI029957, PE02018132), flowering from January to March, fruiting from March to August. Voucher specimens and raw morphological measurements for *S. sinovietnamensis* are summarized in Table S2. Morphological comparison (Table 1) demonstrates that *S. sinovietnamensis* can be distinguished from *S. cocculoides* mainly by its quadrangular stems and the abscission zone at one-third of the petiole length. Most other vegetative and floral morphological characters overlap between the two taxa and are not sufficient for reliable species discrimination. Nevertheless, our phylogenetic analyses support *S. sinovietnamensis* as an independent evolutionary lineage.

### 2.2. Distribution and Habitat

Napo County and Jingxi City, under the jurisdiction of Baise City in the Guangxi Zhuang Autonomous Region of China, are special geographical regions on China’s southwestern border, falling into the category of “frontier-ethnic minority-mountainous-border” type areas. These two places not only boast a long border line and terrain dominated by mountains but also serve as concentrated residential areas for ethnic minorities. Meanwhile, located in the transition zone from the Yunnan-Guizhou Plateau to the western Guangxi hills, they are typical karst landform areas on the southwestern border [21]. In terms of ecological and geomorphic protection, designated exclusive protected areas have been clearly established; their high-quality natural conditions, including karst landforms, primeval forests and rich biodiversity, possess extremely high research and conservation value.

**Figure 3:**
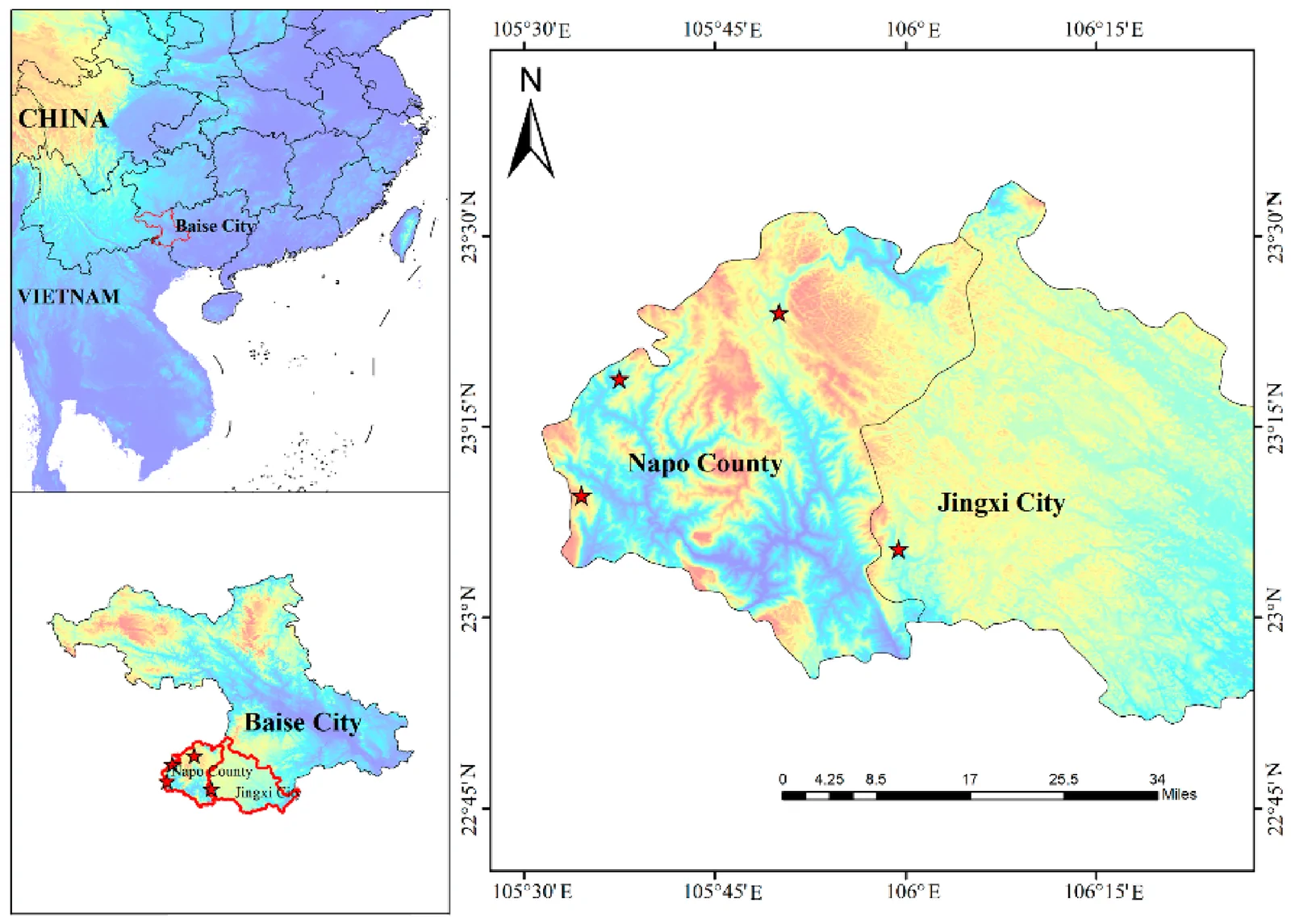
Distribution records of *S. sinovietnamensis* P. Li & X.J. Jin sp. nov. (red stars) from Napo County and Jingxi City, Guangxi Zhuang Autonomous Region, China.

This new species is known from Napo County and Jingxi City, Baise City, Guangxi Zhuang Autonomous Region, China. To date, it has been recorded from one locality in Napo County and one locality in Jingxi City (Figure 3).

### 2.3. Stem Anatomical Characteristics

Transverse-section observations of mature stems of *S. sinovietnamensis* revealed a distinctly quadrangular stem outline, consistent with the gross-morphological feature of four-angled stems observed in field-collected materials (Figure S1A). The outermost layer consisted of compact uniseriate epidermis. Beneath the epidermis was the cortical zone composed of several layers of relatively small parenchyma cells; cortical tissues were obviously thicker at four angular positions, producing the quadrangular contour of the stem cross-section (Figure S1B). Numerous vascular bundles were scattered within large-celled ground parenchyma in the central region, showing typical monocotyledonous atactostele. Each vascular bundle was enveloped by a well-developed sclerenchymatous bundle sheath, with xylem positioned internally and phloem peripherally. No continuous sclerenchyma ring was detected in the cortex. Ground tissue was mainly composed of large thin-walled parenchyma cells, some of which contained dark inclusions. Collectively, the quadrangular stem morphology of *S. sinovietnamensis* is mainly derived from local proliferation and expansion of cortical parenchyma at four corners, rather than modifications to the internal arrangement of vascular bundles.

### 2.4. Phylogenetic Analysis

Based on the concatenated aligned dataset of nrDNA, cpDNA, and ONGs—which encompasses 37 taxa of the Smilacaceae—phylogenetic analyses were performed using two different phylogenetic methods: Bayesian inference (BI) and maximum likelihood (ML). The results showed that all six phylogenetic trees generated by the two methods (Bayesian inference, BI; maximum likelihood, ML) consistently indicated that *S. sinovietnamensis* is nested within Clade C (marked in green in the figures), and it is a sister species to *S. cocculoides* yet exhibits significant genetic differentiation, forming closely related but independent branches. Notably, the sister-group relationship between them obtained high support, with a Bayesian posterior probability (BPP) of 1.0 and a maximum likelihood bootstrap support (ML-BS) of 98.

**Figure 4:**
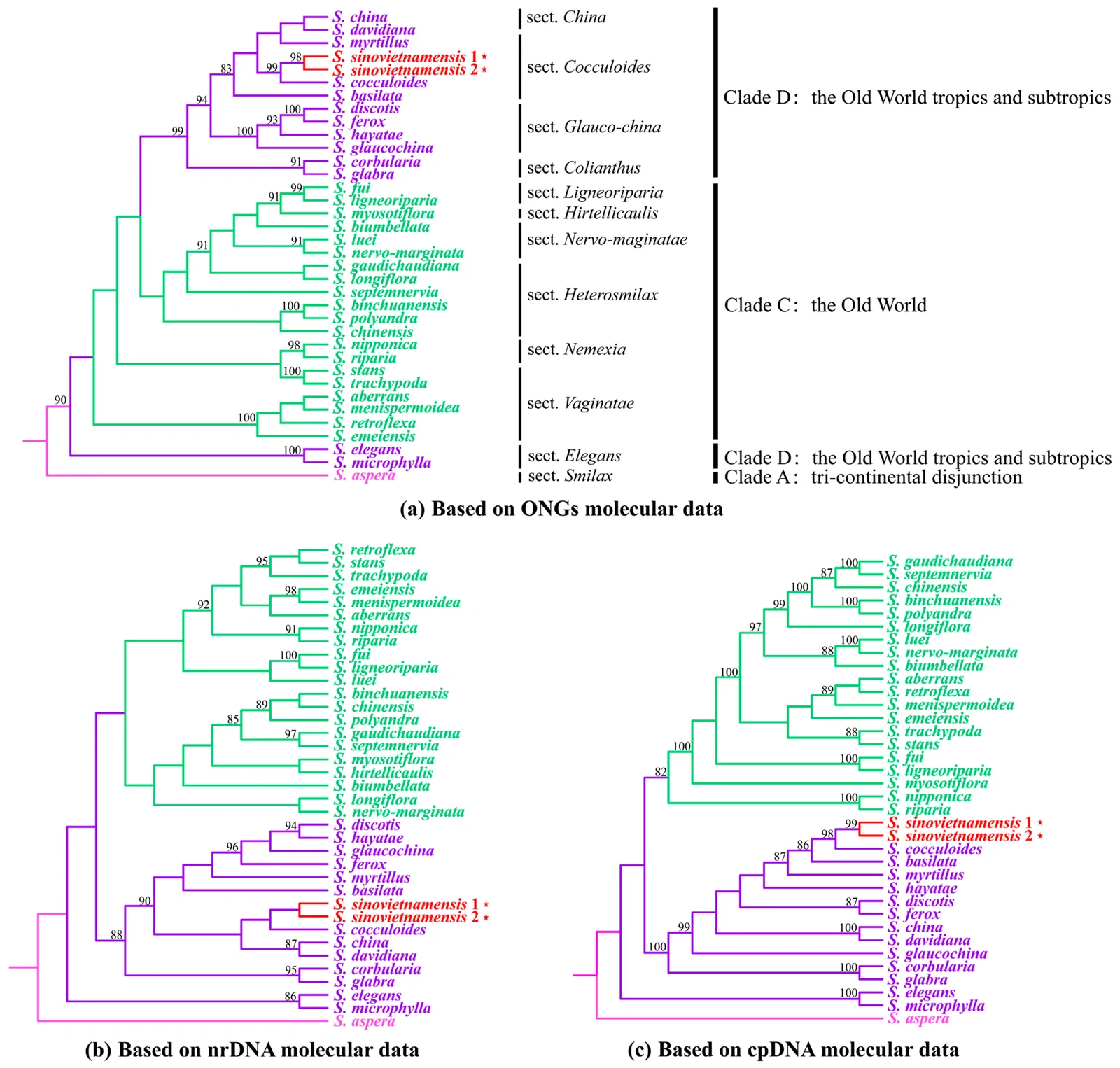
Maximum-likelihood (ML) phylogenetic hypotheses for *Smilax* inferred from aligned datasets of 37 taxa: (**a**) orthologous nuclear genes (ONGs: *At103* and *Agt1*); (**b**) nuclear ribosomal DNA (nrDNA: ITS1, ITS2, 5.8S, 26S); and (**c**) chloroplast DNA (cpDNA: *ndhA*, *ndhF*, *rpl16*, *matK*, *rbcL*). The 37 *Smilax* taxa are grouped into three major clades: Clade A (pink), Clade C (green), and Clade D (purple). Red-marked labels denote the two sampled individuals of our target species *S. sinovietnamensis* in this study. Numbers above branches are ML bootstrap values (ML-BS) > 80%.

**Figure 5:**
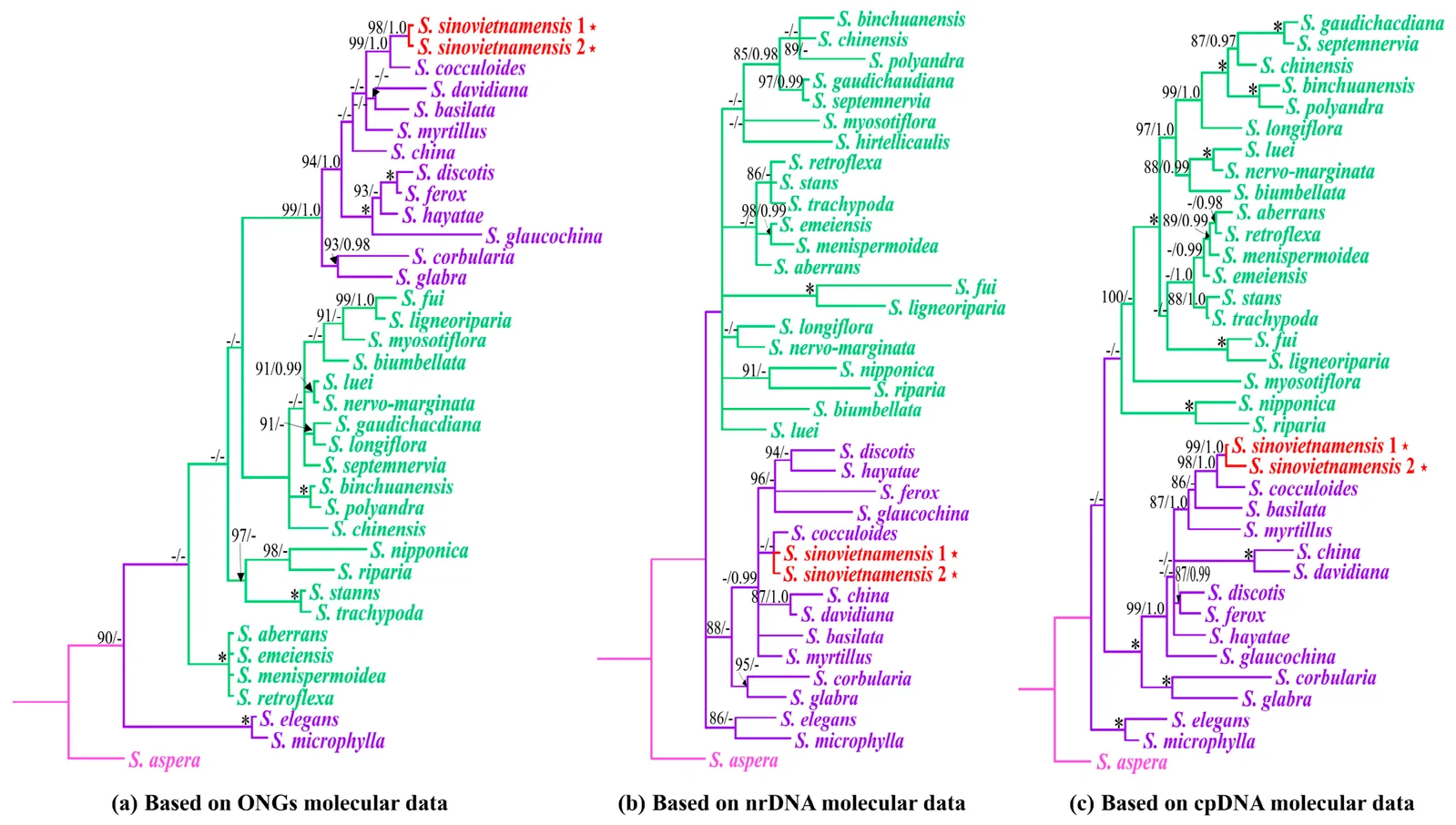
Three Bayesian-inference (BI) phylogenetic hypotheses for *Smilax* based on aligned datasets from 37 taxa: (**a**) orthologous nuclear genes (ONGs: *At103* and *Agt1*); (**b**) nuclear ribosomal DNA (nrDNA: ITS1, ITS2, 5.8S, 26S); and (**c**) chloroplast DNA (cpDNA: *ndhA*, *ndhF*, *rpl16*, *matK*, *rbcL*). The 37 taxa are resolved into three major clades: Clade A (pink), Clade C (green), and Clade D (purple). Numbers above branches show ML bootstrap values (ML-BS) and Bayesian posterior probabilities (BPP). Red-marked labels denote the two sampled individuals of our target species S. sinovietnamensis in this study. An asterisk (*) indicates ML-BS = 100% and BPP = 1.0, and a dash () indicates ML-BS < 85% and BPP < 0.95.

From the analysis of different molecular data, distinct characteristics emerged while maintaining core consistency. Based on ONG molecular data, *S. sinovietnamensis* and *S. cocculoides* formed a highly supported closely related branch (BPP = 1.0, ML-BS = 99). Based on cpDNA molecular data, the support rates (both BI and ML) were ≥95, which not only confirmed the new species’ closest affinity with *S. cocculoides* but also clearly revealed differentiation between *S. sinovietnamensis* 1 and 2 (sampled from different locations)—a differentiation supported by BPP = 1.0 and ML-BS = 99. Additionally, the cpDNA tree showed slight differences from nrDNA and ONG data, reflecting the divergence in evolutionary rates between cytoplasmic and nuclear inheritance. In contrast, based on nrDNA molecular data, the branch support rate between the *S. sinovietnamensis* and *S. cocculoides* was relatively low, only providing a preliminary indication of their close relationship without sufficient support strength (Figure 4 and Figure 5).

### 2.5. Characteristics of the Smilax sinovietnamensis Plastome

The full lengths of the plastomes of *S. sinovietnamensis* 1 and 2 are 158,518 bp and 158,500 bp, respectively. Both plastomes exhibit a typical tetrad structure, which consists of a large single copy (LSC) region, a small single copy (SSC) region, and two inverted repeat (IR) regions. The overall GC content of the *S. sinovietnamensis* plastome is 37.1%, and the GC content of the LSC, SSC and IR regions is 35.1%, 30.9%, and 42.4%. A total of 124 genes are included in the genome (82 protein-coding genes, 6 rRNA genes, and 36 tRNA genes). A total of 15 genes had two copies, including 6 protein-coding genes (*ndhB*, *rpl2*, *rpl23*, *rps7*, *rps19*, *ycf2*), 6 transfer RNA (tRNA) genes (*trnA-UGC*, *trnH-GUG*, *trnL-CAA*, *trnN-GUU*, *trnR-ACG*, *trnV-GAC*), and all three ribosomal RNA (rRNA) species (*rrn16S*, *rrn23S*, *rrn5S*). The *trnE-UUC* gene had three copies, while the *trnM-CAU* gene had four copies. In the genome, six protein-coding genes (*rps16*, *atpF*, *rpoC1*, *rpl2*(×2), *ndhA*) had one intron, and *ycf3* and *clpP* genes contained two introns.

However, there are slight differences in plastome structure and length between *S. sinovietnamensis* 1 and 2 collected from different geographical distribution areas. Specifically, the plastome of *S. sinovietnamensis* 1 (GenBank Accession No. SRR3587995) sampled from Napo County has a total length of 158,518 bp, consisting of a large single-copy (LSC) region of 85,576 bp, a small single-copy (SSC) region of 18,550 bp, and two inverted repeat (IR) regions each of 27,196 bp. In contrast, the plastome of *S. sinovietnamensis* 2 (GenBank Accession No. SRR3587994) collected from Jingxi City has a total length of 158,500 bp. Its LSC region length is consistent with that of *S. sinovietnamensis* 1 (85,576 bp), while the SSC region is 18,576 bp in length and each IR region measures 27,174 bp. Further quantitative analysis revealed the specific length variations in each plastome region between *S. sinovietnamensis* 1 and 2: the total genome length differs by 18 bp; the SSC region length shows a difference of 26 bp; and the length of each IR region differs by 22 bp.

**Figure 6:**
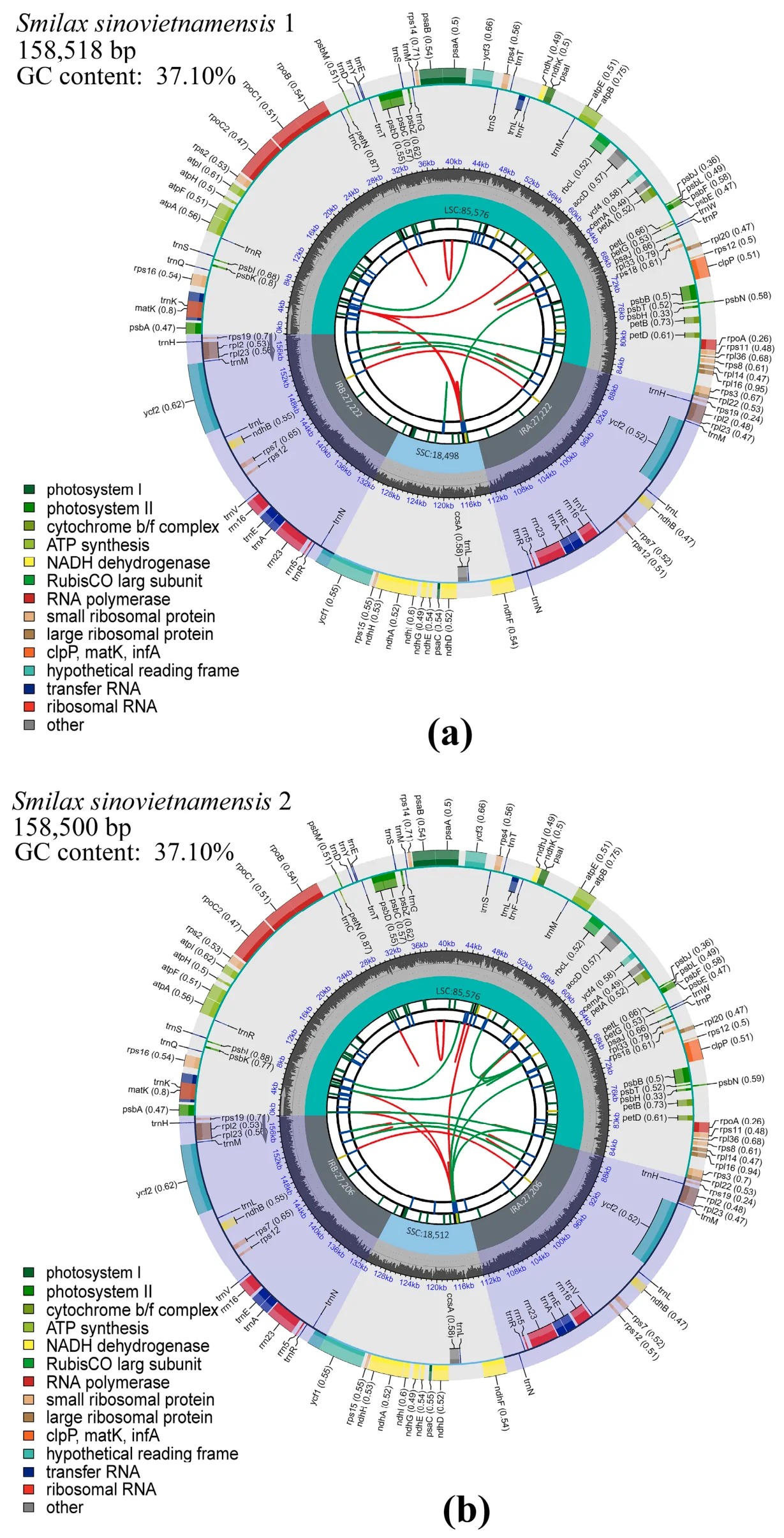
Circular gene maps of the *S. sinovietnamensis* plastome: (**a**) plastome of *S. sinovietnamensis* 1; (**b**) plastome of *S. sinovietnamensis* 2. Genes drawn within the circles are transcribed clockwise, while those drawn outside are transcribed counterclockwise. Genes are color-coded according to their functional groups, and the colored bars indicate genes belonging to different functional groups, and the grey arrows show the transcription direction of corresponding gene clusters.

Differences were also observed in the boundaries of the IR regions between *S. sinovietnamensis* 1 and *S. sinovietnamensis* 2. Specifically, the boundary between the IRa region and the SSC region of *S. sinovietnamensis* 1 showed a cross-region spanning of the *ndhF*, whereas no such spanning was detected at the corresponding boundary (IRa/SSC) of *S. sinovietnamensis* 2 (Figure 6).

## 3. Discussion

### 3.1. Morphological Characteristics of Quadrangular Stems

This study revealed that *S. sinovietnamensis* possesses the trait of a quadrangular stem, which not only distinguishes it from most other species in the Smilacaceae [6] but also represents a character of significant evolutionary importance. Stem-anatomical observations (Figure S1) indicated that the quadrangular outline arises from local proliferation and expansion of cortical parenchyma at the four angular positions rather than from collenchyma-reinforced ridges or outward protrusion of vascular bundles. In the plant kingdom, the stems of the vast majority of species are cylindrical, while only a small number of taxa—such as members of the Lamiaceae [22] and Scrophulariaceae [23]—develop quadrangular stems. Notably, the quadrangular stems of Lamiaceae are formed by collenchyma concentrations along stem ridges, a developmental pathway different from that observed in *S. sinovietnamensis*. Even within the same family, both quadrangular and cylindrical stems may coexist, as exemplified by *Chimonobambusa quadrangularis* in the Poaceae [24]. This specialized morphological trait not only serves as a key diagnostic character in plant taxonomy but also correlates closely with plant growth habits [25]. For instance, *S. elegantissima* [26], *S. gagnepainii* and *S. guiyangensis* in the Smilacaceae are all erect shrubs with quadrangular stems, whereas climbing species within this family possess terete stems [6].

Although the cellular origin of the quadrangular contour in *S. sinovietnamensis* differs from that of typical dicot quadrangular-stemmed groups, this external four-sided morphology may still improve the mechanical properties of the stem, enhancing flexural rigidity and resistance to deformation [22,27]. This provides a plausible explanation for the divergence of growth habits within Smilacaceae: most climbing vines in this family adopt cylindrical stems, which may help to reduce wind-loading; by contrast, the erect shrub *S. sinovietnamensis* may rely on its quadrangular stem to achieve better mechanical self-support. The quadrangular stem could decrease sway amplitude under wind loading and mitigate wind impact, which is advantageous for its erect growth in forest understory habitats. From the perspective of growth-strategy economics, the mechanical reinforcement associated with quadrangular stems in erect shrubs and the relatively flexible cylindrical stems of climbing vines may represent two alternative adaptive solutions within *Smilax*, suggestive of convergent morphological adaptation to distinct growth habits.

Some previous studies have proposed that quadrangular stems in certain plants constitute an adaptation to arid environments, whereby modified xylem conduits restrict the spread of embolism and lower the risk of hydraulic failure [28]. This hypothesis provides an interesting reference for *Smilax*. *S. sinovietnamensis* occurs on limestone outcrops where soil water availability is often limited; other quadrangular-stemmed erect *Smilax* species (*S. elegantissima*, *S. gagnepainii*, *S. guiyangensis*) also occupy comparable rocky habitats [6]. However, our anatomical observations did not detect obvious specialization of xylem structure within the stem. Accordingly, it remains unclear whether the quadrangular morphology itself confers improved drought tolerance. We therefore hypothesize that the quadrangular stem may potentially be an adaptive feature for surviving periodically water-limited limestone habitats, but further physiological and anatomical investigations are required to test this conjecture.

### 3.2. Phylogenetic Tree and Plastome

The six phylogenetic trees constructed based on three types of molecular data (nrDNA, cpDNA, and ONGs) all consistently support that *S. sinovietnamensis* is the sister taxon of *S. cocculoides*.

While clarifying the taxonomic status and intrageneric affinities of *S. sinovietnamensis*, this study also observed a phenomenon: topological discrepancies exist among phylogenetic trees constructed using different molecular markers, accompanied by the occurrence of nuclear-plastid conflict. This phenomenon is not unique to the Smilacaceae but is widely prevalent in the plant kingdom. For instance, in Asteridae, nuclear gene-based phylogenetic trees indicate that Ericales and Cornales form a sister group, and Aquifoliales belongs to Lamiidae; however, plastid gene-based trees place Ericales as the sister group to Gentianidae and assign Aquifoliales to Campanulidae, resulting in distinct divergences in the phylogenetic positions of these taxa [29]. Similar conflicts have also been documented in Orchidaceae: nuclear gene analyses support Vandeae as the sister group to Epidendreae + Cymbidieae, and *Cattleya* as closely related to *Oncidium*; in contrast, plastid gene trees cluster Vandeae with Cymbidieae and group *Cattleya* with *Phalaenopsis*, with strongly divergent phylogenetic placements of genera such as *Coelia* and *Earina* between nuclear and plastid trees [30]. Cytonuclear discordance primarily stems from the independent inheritance of nuclear and cytoplasmic genes, and biological processes such as incomplete lineage sorting (ILS), introgression, and hybridization further amplify the differences arising from this genetic independence, ultimately leading to topological inconsistencies between nuclear gene trees and cytoplasmic gene trees [31]. Such inherent disparities often lead to inconsistencies in the topological structures of phylogenetic trees. Prior phylogenetic analyses of the genus *Smilax* have also reported similar topological discrepancies. For example, Feng et al. [32] observed nuclear-plastid conflict in their study of *S. weniae* when constructing phylogenetic trees based on ITS (a nuclear gene marker) and cpDNA (a plastid gene marker), further confirming the prevalence of this conflict within *Smilax*.

In our nrDNA phylogeny, the clade comprising *S. sinovietnamensis* and its close congener *S. cocculoides* received only weak bootstrap support. Several evolutionary scenarios may account for this poor nodal support. First, historical hybridization and introgression, for which plastid capture has been hypothesized within *Smilax*, could blur phylogenetic signals among closely related species [33]. Second, incomplete lineage sorting (ILS) represents a common source of gene-tree/species-tree discordance during rapid species radiations, where ancestral genetic polymorphisms fail to sort completely among recently diverged lineages [34]. Third, intergenic conversion among paralogous nrDNA copies can homogenize or scramble ITS sequences, eroding the phylogenetic signal for recent species splits [35]. At present, our dataset cannot discriminate among these competing hypotheses. Expanded taxon sampling and multi-locus nuclear genomic data will be required to disentangle the relative contributions of introgression, incomplete lineage sorting, and nrDNA intergenic conversion in this species complex.

In addition to performing phylogenetic analyses, this study also assembled the plastome. Consistent with reports for most angiosperms [36], the plastome of *S. sinovietnamensis* exhibits a typical quadripartite structure, which is congruent with the plastome structures of nine *Smilax* species investigated by Wong et al. [37]. The total length of the plastome of *S. sinovietnamensis* ranges from 158,500 to 158,518 bp, with an LSC region of 85,576 bp, an SSC region of 18,550 bp, and two IR regions each measuring 27,196 bp. Additionally, its GC content is 37.10%, all of which fall within the range of plastome sizes and GC contents reported for *Smilax* species by Wong et al. [37].

However, we observed intraspecific polymorphism specifically at the IRa-SSC boundary between the two geographically separated accessions. In the sample from Napo County, the *ndhF* gene spans the IRa-SSC junction, whereas this cross-boundary pattern was absent in the Jingxi individual. Such positional variation at the IRa-SSC junction falls within the broader phenomenon of IR-SC boundary expansion and contraction, a well-documented dynamic evolutionary phenomenon in angiosperm plastomes, which can be driven by small-scale gene-conversion-mediated drift or large-scale double-strand-break-associated recombination [38]. Similar IR-SC junction variation has also been documented among different species within Smilacaceae [37]. Genes adjacent to these junctions, such as *ndhF* at the IRa-SSC boundary, frequently undergo positional shifts accompanying this kind of boundary mobility. Importantly, this polymorphism represents population-level plastomic variation rather than interspecific divergence. Although the IRa-SSC junction configuration differs between the two populations, the conserved gene content and overall plastome organization still support that these two accessions belong to the same species.

### 3.3. DNA Barcode Genes

The ITS region (a fragment of nrDNA) used in this study has been validated as a DNA barcode by Li et al. [39], exhibiting a significantly higher species discrimination rate than other single molecular markers [39]. ITS is also a widely employed molecular marker in phylogenetic analyses of the Smilacaceae [40]; for example, in the study of *S. weniae*, the ITS sequence was utilized as a molecular marker to construct a phylogenetic tree, which clarified its phylogenetic position within the family. The phylogenetic tree constructed based on ITS in the present study similarly displays a high species discrimination capability. Among the cpDNA fragments selected in this study, *matK* and *rbcL* are internationally recognized DNA barcode genes [39,40], yet their combined species discrimination efficacy remains inadequate [39,40,41]. In previous studies on plants of the family Smilacaceae—*S. weniae*, for instance [32]—a five-marker combination (*ndhA* + *ndhF* + *rpl16* + *matK* + *rbcL*) was established by supplementing three additional genes (*ndhA*, *ndhF*, and *rpl16*) to *matK* and *rbcL*, and this combination was subsequently used for phylogenetic tree construction. In the present study, we also adopted this five-marker combination to construct phylogenetic trees, thereby verifying the novel species status of *S. sinovietnamensis*. The results demonstrated that this five-marker combination exhibits relatively high species discrimination efficacy.

Furthermore, *At103* and *Agt1* employed in this study are categorized as ONGs and belong to the class of conserved orthologous set (COS) markers [42,43]. These markers not only exhibit excellent performance in amplification efficiency, polymorphism level and phylogenetic informativeness, rendering them suitable for phylogenetic reconstruction of closely related plant species but also display robust discriminatory power at both generic and familial taxonomic levels [41,43]. In practical research applications, these two markers were utilized to construct phylogenetic trees and compared against traditional markers such as ITS and *matK*. The results validated their applicability as universal nuclear gene markers for the analysis of closely related species differentiation [41,43]. The phylogenetic trees constructed on the basis of *At103* and *Agt1* in this study further verified their outstanding discriminatory resolution. Therefore, *ndhA*, *ndhF*, *rpl16*, *At103*, and *Agt1* may serve as candidate DNA barcode genes for the genus *Smilax*.

From a comprehensive genetic perspective, this study explores the evolutionary patterns of the Smilacaceae by integrating two types of genetic markers: first, nuclear gene markers reflecting the characteristics of biparental nuclear inheritance, namely ONGs [44] and the ITS [44,45] sequence of nrDNA; second, chloroplast-derived markers representing the pattern of maternal organellar inheritance, including cpDNA [46] and plastomes.

### 3.4. Integrative Approaches to Smilax Taxonomy

In recent years, taxonomic studies of *Smilax* have gradually shifted from reliance on single morphological evidence toward an integrative approach incorporating multiple lines of evidence. Due to the high degree of similarity in overall morphological characteristics among some closely related taxa, species boundaries may be difficult to accurately delimit based solely on morphological traits. Therefore, integrating morphological characteristics with molecular phylogenetic evidence, and further incorporating multilocus nuclear genes, chloroplast markers, and genome-scale data, has become an important approach for improving the reliability of species delimitation in *Smilax*. For example, Feng et al. [32] combined detailed morphological comparisons with cpDNA and nrITS phylogenetic analyses to validate the independent taxonomic status of *Smilax weniae*, demonstrating that the integration of morphological and molecular evidence can effectively support species delimitation within *Smilax*.

However, morphological evidence alone or a single molecular region is often insufficient to fully resolve the complex species relationships within *Smilax*. Morphological traits reflect phenotypic differentiation among species, whereas multilocus molecular markers provide insights into lineage histories from different genetic perspectives. Previous studies on plant DNA barcoding have demonstrated that different molecular markers exhibit distinct advantages and limitations in terms of applicability and discriminatory power and that the combination of multiple markers can improve the identification efficiency of closely related taxa [47]. Therefore, the integration of morphological traits, multilocus nuclear genes, chloroplast molecular markers, and complete plastome data in *Smilax* taxonomy can provide a more comprehensive assessment of species boundaries and evolutionary relationships.

## 4. Materials and Methods

### 4.1. Morphologic Description and Comparison

The morphological data of the new species were collected during field surveys. Specimens were gathered by Pan Li et al. and deposited in the Herbarium of Wenzhou University (WZUH). The multi-dimensional measurement data (including length, size, angle, color, etc.) and morphological details involved in the description are all based on indoor observation results of type specimens and have undergone quantitative processing using the Morphological and Cytotaxonomic Trait Data Analysis Tool MATO _win64_20240920 (Measurement and Analysis Tools) [48]. To further clarify the diagnostic characteristics between *S. sinovietnamensis* and *S. cocculoides*, a comparative analysis of their key morphological traits was conducted simultaneously, specifically including leaf length-width ratio, position of petioles abscission points, texture and surface features of stems, total inflorescence length, pedicel length, number of florets per inflorescence, and length and width dimensions of male and female perianths. Photographs of flowering plants were taken with a Canon (Tokyo, Japan) EOS 6D Mark II digital camera, and the anatomical structures of flowers were observed using an Olympus (Tokyo, Japan) SZ61 stereomicroscope.

### 4.2. Anatomical Observation of Stem

Healthy mature stem segments of *S. sinovietnamensis* were selected for anatomical experiments. The frozen-section technique was adopted for sectioning [49]. An Epredia CryoStar NX50 cryostat was used in this experiment. The specimen-head temperature of the cryostat was set to −12 °C. The stem material was embedded with OCT compound and rapidly frozen on the quick-freezing platform until the embedding medium became fully solidified. After trimming the specimen, formal sections were cut at a thickness of 50 μm. Well-preserved cross-sections were observed under a stereomicroscope and a light microscope, and micrographs were captured for further anatomical analysis [50].

### 4.3. Taxon Sampling and DNA Sequencing

Young fresh leaves of *S. sinovietnamensis* were sampled from the Napo County and Jingxi City. Total genomic DNA was extracted using a modified cetyltrimethylammonium bromide method [51]. The extracted DNA was sheared to fragments shorter than 800 bp, and libraries were prepared with the Illumina Nextera XT kit (Illumina, San Diego, CA, USA). Fragment quality was assessed using a 2100 Bioanalyzer (Agilent, Santa Clara, CA, USA). Short-insert libraries (with an insert size of 350 bp) were sequenced by the Beijing Genomics Institute on an Illumina HiSeq 2500 platform (Illumina, San Diego, CA, USA), yielding 150 bp paired-end reads. High-quality reads of 17 GB were obtained.

### 4.4. Assembly and Annotation of the Plastome, ONGs, cpDNA, and nrDNA

The complete plastomes, nrDNA (ITS1-5.8S-ITS2-26S), cpDNA (*ndhA*, *ndhF*, *rpl16*, *matK*, *rbcL*), and ONG (*At103*, *Agt1*) sequences of *S. sinovietnamensis* were assembled based on clean reads obtained from genome skimming using GeneMiner v20250421 [52]. GeneMiner simultaneously completed the automatic annotation of the plastome, including genes, CDS, tRNA, and rRNA. The positions of the start codon, stop codon, and exon boundaries of all genes were precisely verified and manually adjusted in Geneious v2024.0.5 [53]. The plastome map of *S. sinovietnamensis* was drawn online using OGDRAW v2 (web-based, http://ogdraw.mpimp-golm.mpg.de/, accessed on 24 July 2025) [54]. The final sequences of the whole plastomes, nrDNA, cpDNA, and ONGs of *S. sinovietnamensis* were submitted to GenBank, with the accession numbers listed in Table S1.

### 4.5. Phylogenetic Analyses

In this study, *S. aspera* L. is selected as the outgroup. The sampling scope covers three geographical clades (Clade A: trans-tricontinental disjunct taxa, Clade C: Old World taxa, and Clade D: Old World tropical and subtropical taxa), totaling 37 distinct species of *Smilax.* Among these, the clade containing *S. cocculoides* comprises four species represented by five accessions. To speculate on the phylogenetic position of the new *Smilax* species, phylogenies were reconstructed by maximum likelihood (ML) and Bayesian inference (BI) methods using the three datasets: (a) concatenated sequences of four nrDNA (ITS1, ITS2, 5.8S, and 26S); (b) concatenated sequences of five cpDNA (*ndhA*, *ndhF*, *rpl16*, *matK*, and *rbcL*); (c) concatenated sequences of two ONGs (*At103* and *Agt1*). Furthermore, given the differences in the sources of genetic material among the datasets, each dataset was analyzed separately to construct phylogenetic trees, thereby avoiding nuclear-cytoplasmic conflicts [29]. In all phylogenetic trees, *S. aspera* was selected as the outgroup since *S. aspera* has consistently been considered an early-diverging basal lineage [2,3]. Except for *S. sinovietnamensis*, all nrDNA, cpDNA, and ONG sequences were downloaded from the National Center for Biotechnology Information database (https://www.ncbi.nlm.nih.gov/, last accessed on 26 July 2025). GenBank accession numbers and voucher information of the species used for phylogenetic analysis were provided in Table S1. Each nrDNA, cpDNA, and ONG sequence was aligned, edited, and individually concatenated into a supermatrix using Geneious v2024.0.5 and its associated plugins, such as the Muscle Alignment function and Concatenate Sequences Alignments.

For the ML analysis, IQ-TREE v1.6.8 [55] was utilized, with the “-p” command used to partition the different segments of each dataset. Bootstrap support values were calculated based on 5000 replicates using the “-m” command to find the best-fit models, with the optimal models determined as follows: For chloroplast DNA (cpDNA), the optimal model for *matK*, *ndhA* coding sequences (CDS), and *ndhF* is TVM + F + I; the optimal model for *rbcL* is F81 + F + I + G4; the optimal model for *ndhA* introns is HKY + F + G4; and the optimal model for *rpl16* is F81 + F + I. For nuclear ribosomal internal transcribed spacer and related sequences (ITS), the optimal model for the ITS1 and ITS2 regions is GTR + F + G4, and the optimal model for the ITS, 5.8S, ITS, and 26S regions is HKY + F. For orthologous nuclear genes (ONGs), the optimal model for the coding sequences (CDS) of *Agt1* and *At103* is TPM3 + FQ + I, and the optimal model for the introns of *Agt1* and *At103* is HKY + F + G4. Finally, the generated phylogenetic trees were visualized using Figtree v1.4.4 (http://tree.bio.ed.ac.uk/software/figtree/, accessed on 3 June 2025) and further embellished by Adobe Illustrator v2025 software. Bayesian Inference (BI) was performed using MrBayes v. 3.2.7 [56] via the CIPRES Science Gateway. For the three categories of sequences involved in this study (cpDNA, nrDNA, ONGs), the initial optimal nucleotide substitution models and partitioning schemes (Cprev) were first identified using PartitionFinder-2.1.1 [57] based on the Akaike Information Criterion (AIC), corrected Akaike Information Criterion (AICc), and Bayesian Information Criterion (BIC). The optimal models and partitioning schemes for each gene in MrBayes were finally determined as follows: Specifically, for chloroplast DNA (cpDNA), a partitioning scheme with 2 subsets was adopted—the first and second codon positions of all genes were merged into Subset1 (optimal model: GTR + G), while all third codon positions were merged into Subset2 (optimal model: GTR + G). For nuclear ribosomal DNA (nrDNA), a partitioning scheme with 4 subsets was used: all codon positions of Gene1 constituted Subset1, and all codon positions of Gene2 constituted Subset2 (both with the optimal model GTR + G); Gene3 formed Subset3 (optimal model: JC); and Gene4 formed Subset4 (optimal model: HKY). As for orthologous nuclear genes (ONGs), a “fully merged (allsame)” partitioning scheme was adopted, with the optimal model being HKY + G. In MrBayes, the charset command was used to specify the site ranges of each subset, the partition command to combine the partitions, and the aforementioned lset command (along with the necessary prset command) to assign the corresponding optimal models. Subsequently, the Markov Chain Monte Carlo (MCMC) algorithm was run separately for each dataset, with a total of 10,000,000 generations, 4 independent runs (each with 4 parallel chains), default parameters, and a randomly generated initial tree; tree topologies were sampled every 1000 generations, and convergence of the chains was determined when the average standard deviation of split frequencies (ASDFs) was ≤0.01. After discarding the first 25% of the trees as burn-in for each dataset, the maximum clade credibility tree was constructed based on all remaining sampled trees, and Bayesian posterior probabilities (BPPs) for each clade on the tree were calculated. Finally, the generated phylogenetic trees were visualized using Figtree v1.4.4 (http://tree.bio.ed.ac.uk/software/figtree/, accessed on 10 June 2025) and further embellished by Adobe Illustrator software.

## 5. Conclusions

This study integrates morphological characteristics and multi-locus molecular evidence to formally describe and publish *S. sinovietnamensis*, a new species of Smilacaceae from the Sino-Vietnamese border region. Phylogenetic analyses based on nrDNA, cpDNA, and ONGs reveal significant genetic divergence between this species and its sister taxon *S. cocculoides*. The new species can be clearly distinguished from *S. cocculoides* by its quadrangular stems, robust habit, and abscission zone located at one-third of the petioles’ length. Meanwhile, this study is the first to report the plastome characteristics of this new species, providing new data for resolving the evolutionary history of Smilacaceae. Anatomical data obtained from stem transverse-sectional observations further enriches the morphological evidence supporting the delimitation of this new taxon. Its specialized quadrangular stem is not only a key diagnostic trait but also offers important insights into the adaptive evolution of stem morphology and the morphological diversification of angiosperms. In summary, the integrative evidence confirms the specific status of *S. sinovietnamensis* and provides effective methods and perspectives for addressing taxonomic challenges in the genus *Smilax* and exploring its speciation and adaptation mechanisms.

## Figures and Tables

**Table 1:** Differences between *S. sinovietnamensis* and *S. cocculoides*. Data on *S. cocculoides* are obtained from Flora Reipublicae Popularis Sinicae [20].

Characters	*S. sinovietnamensis*	*S. cocculoides*
Stem	quadrangular stems, significantly thicker stems	cylindric stems, relatively slender stem
Leaf blade	6.8–19 cm long, 2.7–10.6 cm wide, the absence of indented reticulate veins	5–12 cm long, 2.5–4(–6.5) cm wide29
Petiole	an abscission zone at one-third of the petiole length	an abscission zone near the mid-region of the petiole
Tepal	0.8–4.9 mm long, 0.3–2.9 mm wide	2.5–3.5 mm long, 1.5 mm wide

## Data Availability

The molecular data that support the findings of this study are openly available in GenBank (accession numbers are listed in Table S1).

## Supplementary Materials

The following supporting information can be downloaded at: https://www.mdpi.com/article/10.3390/plants15193024/s1, Table S1: Taxa included in the present study with voucher information and GenBank accession numbers for the sequences of internal transcribed spacer (ITS) of nuclear ribosomal DNA, *matK*, *rbcL*, *ndhA*, *ndhF* and *rpl16*. Missing sequences are indicated by a dash (-). Accession numbers in bold are newly generated sequences; Table S2: Specimen vouchers and original measurement data of *Smilax sinovietnamensis*; Figure S1: Transverse sections of mature stem of *Smilax sinovietnamensis*. (A) Overview showing the distinctly quadrangular outline of the stem (scale bar, 0.5 mm). (B) Magnified micrograph of a stem corner region (scale bar, 800 μm), displaying locally thickened cortical parenchyma at the angular position, with scattered vascular bundles of the atactostele visible within the central ground parenchyma.
